# MicroRNA Expression Analysis of Mice Retinas with Oxygen-Induced Retinopathy by RNA Sequencing

**DOI:** 10.1155/2022/9738068

**Published:** 2022-03-03

**Authors:** Xiuping Chen, Xianglian Li, Yan Liu, Yuanzhi Yuan, Yifan Feng, Jing Wang, Min Li, Dongmei Gao, Fei Yuan

**Affiliations:** ^1^Department of Ophthalmology, Zhongshan Hospital of Fudan University, Shanghai, China; ^2^Department of Ophthalmology, Ninth People's Hospital, Shanghai JiaoTong University School of Medicine, Shanghai, China; ^3^Liver Cancer Institute, Zhongshan Hospital of Fudan University, Shanghai, China

## Abstract

**Purpose:**

To characterize the microRNA (miRNA) expression profiles in the retinas of mice with oxygen-induced retinopathy by RNA sequencing and to ascertain miRNAs associated with retinal neovascularization.

**Methods:**

Retina samples were obtained from 3 groups (6 retinas/group) of OIR mice and normal mice at P17. RNA was isolated from 24 retina samples and then detected on an Illumina HiSeq. Twelve retina samples were used for quantitative polymerase chain reaction to validate the RNA sequencing. Bioinformatics analyses were performed.

**Result:**

The RNA sequence showed that 565 miRNAs were detected in the retina of OIR mice and 583 miRNAs in the retina of normal control mice. A total of 553 miRNAs were expressed in both groups. Thirty-eight miRNAs showed altered expression in both groups (*p* ≤ 0.05). Compared with the control group, 2 miRNAs were significantly upregulated in the OIR group, while 36 miRNAs were significantly downregulated. Meanwhile, 2 candidate miRNAs (miR-181a-5p and miR-21a-5p) with significant differences in miRNA expression (*p* < 0.01) were selected for validation. Quantitative real-time polymerase chain reaction (qRT-PCR) was used to confirm the relative expression of the two miRNAs. Bioinformatics analyses showed that pathways involved in ischemic retinopathy (such as TGF-*β*, Ras, Hippo, PI3K-Akt, VEGF, and HIF-1 signaling pathways) were enriched.

**Conclusions:**

Our study provided an overall view of miRNA profiling in the OIR retina. These miRNA profiles provide a valuable framework for the potential therapy of retinal angiogenesis.

## 1. Introduction

Ischemic retinopathy (IR) is defined as abnormal neovascularization of the retina followed by ischemia, which is the most common vision-threatening complication in many diseases, such as retinopathy of prematurity (ROP), proliferative diabetic retinopathy (PDR), and retina vein occlusion (RVO) [1, 2]. Hypoxia alters the microvascular circulation of the retina and causes a series of structural changes, including increased permeability, disruption of vascular integrity, and retinal neovascularization (RNV), and leads to profound alterations in gene expression [3, 4]. The pathogenesis of pathological RNV is multifactorial, such as angiogenesis [5], oxidative stress [6], and inflammation [7], and its underlying pathogenic mechanism is still not fully understood. Accumulating evidence indicates that microRNAs (miRNAs) may be aberrantly expressed and may play vital roles in the development of RNV [8, 9].

MicroRNAs (miRNAs) are endogenous noncoding RNAs with a length of about 22 nucleotides which involve in the posttranscriptional regulation of gene expression [10] and regulate a wide range of physiological and pathological processes [11, 12]. They are key regulators of vessel development [13] and contribute to the formation of pathological RNV [9, 14]. Recent studies have extensively characterized the involvement of miRNA-mediated regulation in retinal angiogenesis [15], proliferation [16], apoptosis, and migration. Alterations in miRNA expression have been studied in different signaling pathways involved in the progression and pathogenesis of RNV. However, whether there is a correlation between retinal miRNAs and the development of retinal neovascularization is largely unknown. Identifying retinal miRNAs differentially expressed genes in RNV may reveal potentially effective miRNAs and help to clarify their pathogenesis and mechanisms.

Currently, the oxygen-induced retinopathy (OIR) [17] mouse model has been widely used in ROP and PDR studies. Mouse pups are exposed to hyperoxia on postnatal day 7 (P7) to obliterate retinal capillaries. Upon return to room air, hypoxia triggers a repair response of retina vessels, which leads to the formation of retinal neovascular tufts. P17 is the peak of pathological NV. Previous studies have revealed different miRNAs expressions in OIR, but they exhibit a high variability of the identified miRNAs [9, 18, 19]. Therefore, in this study, we used RNA sequencing technology to evaluate the miRNA profiles in OIR retinas at P17 compared with the normal retinas and to investigate which miRNA expression was altered. Alterations in miRNA expressions were validated by qRT-PCR. The upregulation and downregulation of miRNAs may provide an improved understanding of the mechanism of ischemic retinopathy.

## 2. Materials and Methods

### 2.1. Establishment of an Oxygen‐Induced Ischemic Retinopathy Mouse Model

An OIR mouse model was induced as previously described [20]. On P7 after birth, C57BL/6J mice were exposed to 75% oxygen with their nursing mother for 5 days and then returned to normal air (∼21% oxygen) on P12. Age‐matched control mice were kept in normal air. Retinal samples were collected on P17.

### 2.2. Flat‐Mount Retina

OIR and control mice were euthanized at P17. Eyes were enucleated and then fixed with 4% paraformaldehyde for 4 hours. Retinas were collected and blocked with 0.1% Triton X‐100 and 0.5% bovine serum albumin in PBS for 1 hour. After washing in PBS for three times, the retinas were stained with Dylight 594 isolectin B4 for 45 min, then cut into 4–6 radial petals, and flat‐mounted with a fluorescence mounting medium (DAKO; Agilent Technologies, CA). Images were acquired using a confocal microscope.

### 2.3. RNA Extraction and Sequencing Analysis

On P17, 18 OIR mice and 18 normal mice were divided into 3 groups. The retinas were collected for RNA extraction. RNA isolation was performed as described previously [15]. In brief, total RNA was extracted from OIR and normal retina samples using TRIzol reagent (Thermo Fisher Scientific, Waltham, MA). MiRNeasy Kit (Qiagen, Germantown, MD) was used to prepare an RNA sequencing library. RNA sequencing was performed on an Illumina HiSeq 2000 sequencing system (Illumina, San Diego, CA).

### 2.4. Quantitative Real‐Time PCR (qPCR)

According to the manufacturer's instructions, total RNA was isolated from retinas using TRIzol reagent (Thermo Fisher Scientific, Waltham, MA). Total cDNA was synthesized using a miRNA first-strand cDNA synthesis kit (Sangon Biotech, Shanghai, China). Real‐time qPCR was performed using a miScript SYBR Green PCR Kit (Qiagen) and a LightCycler 480 Real‐Time System (Roche, Mannheim, Germany). U6 small nuclear RNA was used as an internal control. Expression levels were quantified using the 2^−ΔΔCt^ method [21].

### 2.5. Bioinformatics Analyses

We conducted gene ontology (GO) analysis (https://www.geneontology.org) and Kyoto Encyclopedia of Genes and Genomes (KEGG) pathway function enrichment analysis (https://www.genome.jp/kegg/) to predict the possible function of those target genes of altered miRNAs.

### 2.6. Statistical Analysis

Comparison between the two groups was performed using Student's *t*-tests. We calculated the *p* values of each miRNA between OIR and control retinas. All statistical analyses were conducted using SPSS 22.0 software (Chicago, IL). *p* < 0.05 was considered statistically significant.

## 3. Results

### 3.1. Successful Establishment of the OIR Model

**Figure 1:**
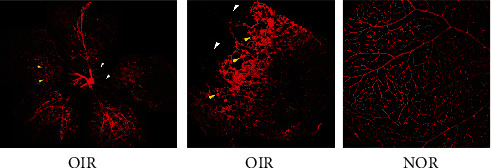
Flat-mount retina image of OIR and NOR. The avascular area (white arrows) and neovascular tufts (yellow arrows) were evident on the OIR retina compared with the NOR retina. The retinal vessels in NOR were normal.

To understand the role of miRNA in ischemic retinopathy, we induced a mouse model of oxygen‐induced ischemic retinopathy. On the whole flat-mount image of the retina, the avascular area around the optic papilla and a large number of neovascular tufts can be observed (Figure 1) compared with the NOR retina, which verifies the successful establishment of the OIR model.

### 3.2. miRNA Expression Profiles in OIR and Normal Mouse Retinas

**Figure 2:**
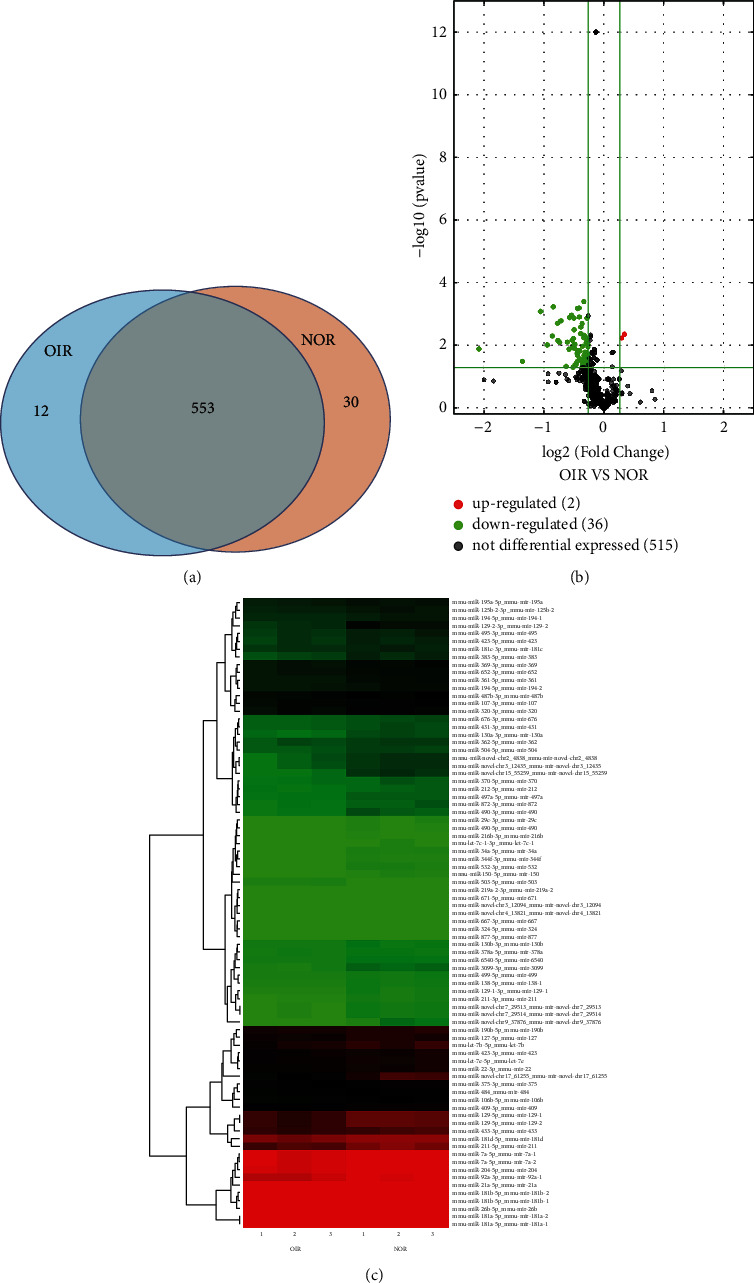
(a) Venn diagram of microRNA (miRNA) detected by RNA sequencing. A total of 565 miRNAs were detected in the OIR mouse retina, and 583 miRNAs were detected in the normal group. A total of 553 miRNAs were expressed in both groups. (b) Volcano plots showing dysregulated miRNAs in the OIR group vs. normal group. Compared with the normal group, the expression of 2 miRNAs was significantly upregulated and the expression of 36 miRNAs was significantly downregulated in the OIR group. (c) Heat map of miRNAs expressed at different levels in OIR retinas vs. normal retinas (6 retinas/group). Red indicates a relatively higher expression, and green indicates a lower expression.

To identify the miRNA expression in OIR and normal groups, we employed RNA sequencing technology. Each retinal sample contained an average of 45.70 ± 13.56 *μ*g RNA (range: 28–63 *μ*g; Supplementary Table 1). We detected 565 miRNAs in the OIR mouse retina and 583 miRNAs in the normal group. In total, 595 expressed miRNAs were identified in both groups (Figure 2(a)). miRNAs expressed at different levels in OIR retinas and normal retinas are shown in Figure 2(c). Among the most highly expressed miRNAs, miR-183-5p, miR-182-5p, let-7i/g/f, miR-26a-5p, miR-148a-3p, miR-30a-5p, miR-96-5p, miR-181a-5p, miR-9-5p, miR-30c-5p, and miR-340-5p were present in both groups. The miRNA with the highest expression in both groups was miR-183-5p.

### 3.3. Identification of Differentially Expressed miRNAs between OIR and NOR Groups

Relative to the NOR groups, 38 miRNAs were modulated by >25% in OIR groups (*p* ≤ 0.05, Figure 2(b)). However, compared with the NOR groups, two miRNAs were significantly upregulated and 36 miRNAs were downregulated in OIR groups. Among the 36 miRNAs downregulated, three miRNAs (miR-129-2-3p, miR-3099-3p, and miR-150-5p) were the most prominently downregulated. Details are shown in Tables 1 and 2.

### 3.4. Target Gene Prediction of Differentially Expressed miRNAs

**Figure 3:**
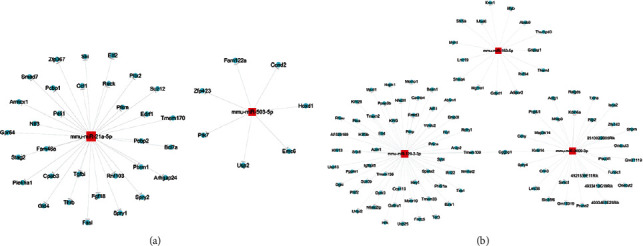
Target prediction network of upregulated and downregulated microRNAs in the OIR retina. (a) The target prediction network of 2 upregulated miRNAs (mmu-miR-21a-5p and mmu-miR-503-5p) in the OIR retina. (b) The target prediction network of 3 downregulated miRNAs (including mmus-miR-150-5p, mmu-miR-129-2-3p, and mmu-miR-3099-3p) in the OIR retina.

The target prediction network of 2 upregulated miRNAs (mmu-miR-21a-5p and mmu-miR-503-5p) in the OIR retina is shown in Figure 3(a). The target prediction network of 3 downregulated miRNAs (including mmus-miR-150-5p, mmu-miR-129-2-3p, and mmu-miR-3099-3p) in the OIR retina is shown in Figure 3(b). The network showed the direct interaction between miRNAs and target genes that may respond to hypoxia and angiogenesis in the OIR group.

### 3.5. Bioinformatics Analysis

**Figure 4:**
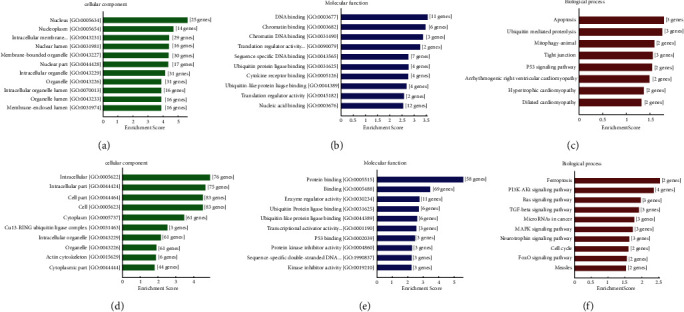
(a) Cellular component analysis of upregulated miRNAs. (b) Molecular function analysis of upregulated miRNAs. (c) Biological process analysis of upregulated miRNAs. (d) Cellular component analysis of downregulated miRNAs. (e) Molecular function analysis of downregulated miRNAs. (f) Biological process analysis of downregulated miRNAs.

To comprehensively analyze the cellular components, molecular functions, biological process, and pathways affected by these dysregulated miRNAs, we performed GO and KEGG pathway enrichment analyses. Enrichment scores of Gene Ontology analyses of differentially expressed miRNAs in the OIR retina are shown in Figure 4. Top KEGG pathways (ranked by enrichment scores) after analyzing 2 upregulated miRNAs are shown in Figures 4(a)–4(c) and Table 3. The top 10 KEGG pathways (ranked by enrichment scores) after analyzing 36 downregulated miRNAs are shown in Figures 4(d)–4(f) and Table 4.

### 3.6. Verification of miRNAs Profiling by qRT-PCR

**Figure 5:**
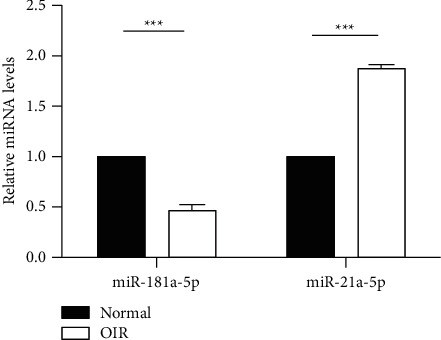
Real‐time qPCR detected the relative expression of miR‐181a‐5p and miR‐21a‐5p in OIR and NOR groups (*n* = 3/group; Student's *t*-tests).

To validate the microRNA expression in our sequencing results, we chose to use qRT-PCR to detect two of the most significantly dysregulated miRNAs in sequencing, miR-21a-5p (upregulated miRNA) and miR-181a-5p (downregulated miRNA). The expression level of miR-21a-5P detected by qRT-PCR was significantly higher in the OIR group than in the NOR group (*p* ≤ 0.01). Inversely, the expression level of miR-181a-5P was significantly lower in the OIR group than in the NOR group as shown in Figure 5, in accordance with the RNA sequencing results.

## 4. Discussion

miRNAs play a key role in cell function and biological development, such as neurogenesis, metabolism, inflammation, and angiogenesis [22]. Recent studies have reported the dysregulation of miRNA in retinal neovascularization [23, 24]. Further describing the role of miRNA may be potentially effective miRNA-based therapeutics for RNV. In this study, we demonstrated the differential expression profiles of miRNA in the OIR model by RNA sequencing analyses and bioinformatics analyses. We investigated the comprehensive miRNA profiles in the OIR retina and the putative roles of the miRNAs. Analyzing using a Venn diagram, we found the miRNA expression differences between OIR and NOR retinas. A total of 565 miRNAs were detected in the OIR retinas and 583 miRNAs in NOR retinas. A total of 595 miRNAs were expressed in both groups. Among the miRNAs with the highest expression, miR-183-5p, miR-182-5p, let-7i/g/f, miR-26a-5p, miR-148a-3p, miR-30a-5p, miR-96-5p, miR-181a-5p, miR-9-5p, miR-30c-5p, and miR-340-5p were found in both groups. The most expressed miRNAs were miR-183-5p in both groups. Some previous studies have explored the role of miRNAs in RNV. The expression profile in the retina and choroid of OIR has been reported using Next-Generation Sequencing (NGS), and miR-9a-5p and miR-182 were reported as the most abundant miRNAs expressed in both the retina and choroid, consistent with our results [25].

Subsequently, we identified 38 miRNAs as OIR-responsive miRNAs by examining the significantly altered expression. A total of 38 miRNAs were differentially expressed between the two groups (*p* ≤ 0.05). Compared with NOR groups, 2 miRNAs were significantly upregulated and 36 miRNAs were significantly downregulated in OIR groups. Among the downregulated miRNAs, miR-129-2-3p, miR-3099-3p, miR-150-5p, miR-181a-5p, miR-129-5p, let-7b-5p, miR-383-5p, miR-369-3p, miR-375-3p, and miR-211-3p rank in the top 10. Among these miRNAs, some have proved target genes involved in pathways with important roles in regulating angiogenesis. Previous studies have shown that miRNA-129-2-3p repressed the proliferation and invasion of intrahepatic cholangiocarcinoma cells [26]. The role of miR-181a-5p has been described in our previous study [15]. Overexpression of miR-181a-5p inhibits retinal neovascularization, which is consistent with other studies [27, 28] and our RNA sequencing results. Jiang's report [29] revealed that miR-129-5p is highly expressed in prostate cancer tissues and overexpression of miR-129-5p significantly reduces cell migration, invasion, and angiogenesis. Overexpression of miR-383-5p inhibits the proliferation and migration of gastric cancer cells [30]. Pan's report [31] found that upregulation of miR-369-3p suppresses cell migration and proliferation in Hirschsprung disease. Yuan's report [32] revealed that miR-375-3p regulates pulmonary microvascular endothelial cell proliferation, chemotaxis, angiogenesis, and inflammation by targeting Notch I. Endothelial miR-150 targets angiogenic genes, such as Fzd4 and DII4 [33], suggesting miR-150 as an intrinsic inhibitor of RNV. Based on these known functions, our findings indicate that miR-3099-3p and let-7b-5p may also participate in RNV neovascularization. However, the role of other dysregulated miRNAs remains unknown such as miR-487b-3p and miR-320-3p, indicating that they may be involved in retinal angiogenesis. As we know, the main function of miRNAs is to silence the expression of the target genes through translational repression or mRNA degradation [34]. Therefore, the downward trend of overall retinal miRNA expression derived from other studies is consistent with our findings.

In our study, we focused on the proliferative phase of OIR with neovascularization. The retinal samples collected at P17 showed the largest pathological new vessels [35]. Early studies explored the miRNA profiles in OIR, suggesting that many miRNAs are involved in retinal neovascularization [9, 18, 19, 36, 37]. There are partial overlaps among the differentially expressed miRNAs. Compared with Zhang's report, several miRNAs including miR-383-5p, let-7c-5p, and let-7e-5p showed a consistent downregulation pattern in our study [36]. miR-495-3p, miR-130a-3p, miR-872-5p, and miR-181c-3p are found significantly downregulated in our study, in agreement with Wang's report [18]. According to our results, miR-129-3p, miR-375-3p, miR-150-5p, miR-383-5p, and miR409-3p were found to be significantly downregulated, which is consistent with Liu's report [37]. In our study, we found that miR-150-5p was significantly downregulated, which is consistent with the report of Shen conducted using microarrays [9]. miR-150 deletion significantly increased retinal pathological angiogenesis in type 2 diabetic mice induced by a high-fat diet, which was partly via VEGFR2 [38]. miR-383-5p was downregulated in three studies. It was reported that miR-383 inhibits the apoptosis of retinal pigment epithelial cells in diabetic retinopathy [39] and inhibits proliferation, migration, and angiogenesis of endothelial cells exposed to gliomas through VEGF-mediated FAK and Src signaling pathways [40]. In contrast, miR-211 was identified in two independent studies, but in opposite directions. Our study showed that miR-211 was downregulated in the OIR retina, while another study showed that it was upregulated in the OIR retina [41]. We postulated that the inconsistent results may be caused by different experimental settings and different animal models. Different miRNAs were found in our study but were not reported in other studies. Further work is needed to find other potential targets of each of these three microRNAs and to precisely define the targets and mechanisms of all microRNAs identified in our research.

To validate our RNA sequencing results, we performed qRT-PCR to verify one upregulated miRNA and one downregulated miRNA as the most abundant in sequencing, miR-21a-5p (upregulated miRNA) and miR-181a-5p (downregulated miRNA). The expression level of miR-21a-5P detected by qPCR was significantly higher in the OIR group than in the NOR group (*p* ≤ 0.01). Inversely, the expression levels of miR-181a-5P detected by qPCR were significantly lower in the OIR group than in the NOR group, in accordance with our RNA sequencing results. Sun [27] reported that overexpression of miR-181a/b-5p reduced cellular proliferation, migration, invasion, and tube formation. Li [28] reported that miR-181a-5p decreased the migration and invasion of cancer cell by targeting the matrix metalloproteinase-14. Liu [42] reported that overexpression of miR-21a-5p promoted fibroblast activity of spinal fibroblasts after mechanical trauma and enhanced the proliferation of spinal fibroblasts.

miRNAs exert their functions through downstream targets. Therefore, we comprehensively analyzed the cellular components, molecular functions, biological processes, and pathways affected by these dysregulated miRNAs, and we performed the enrichment analysis of GO and KEGG pathways. Among the upregulated miRNAs, the top five enriched CCs included nucleus, nucleoplasm, intracellular membrane-bounded organelle, nuclear lumen, and membrane-bounded organelle, while the top five MFs were DNA binding, chromatin binding, chromatin DNA binding, translation regulator activity, and sequence-specific DNA binding. The top five enriched BPs (ranked by enrichment scores) were apoptosis, ubiquitin-mediated proteolysis, mitophagy, tight junction, and p53 signaling pathway. Among the downregulated miRNAs, the top five enriched CCs included intracellular, intracellular part, cell part, cell, and cytoplasm. The top five MFs were protein binding, binding, enzyme regulator activity, ubiquitin protein ligase binding, and ubiquitin-like protein ligase binding. The top five enriched BPs (ranked by enrichment scores) were ferroptosis, PI3K-Akt signaling pathway, Ras signaling pathway, TGF-*β* signaling pathway, and microRNAs in cancer. Other pathways include Hippo signaling, MAPK signaling pathway, and FoxO signaling pathway. The Hippo signaling pathway is an evolutionarily conserved signaling cascade that regulates cellular proliferation and organ size [43]. It is controlled by the phosphorylation of the downstream transcriptional coactivator Yes-associated protein, which could bind to the TEA domain transcription factor to stimulate angiogenesis of ECs [44]. In addition, the VEGF signaling pathway and hypoxia-inducible factor-1a, which are important for retinal angiogenesis, are also among the significantly involved pathways. The possible pathways regulated by these differentially expressed miRNAs include PI3K-Akt, TGF-*β*, and MAPK signaling pathways. In future studies, the genes and signaling pathways targeted by miRNAs should be further explored. Therefore, our prediction is that differentially expressed miRNAs have the ability to target multiple components in these key pathways, which makes them promising molecular targets for RNV therapy.

In summary, our study elucidated the expression pattern of miRNAs in the OIR mouse model. We presented differentially expressed miRNAs and new miRNAs that may be involved in ischemic retinopathy (miR-3099-3p, let-7b-5p, miR-487b-3p, and miR-320-3p) and proposed miRNAs with different expressions (high or low) compared to previous studies (miR-211). In addition, among the differentially expressed miRNAs, we identified two miRNAs (miR-181a-5p and miR-21a-5p) that were significantly and robustly dysregulated as the most abundant in sequencing. Injections or other interventions to ameliorate the reduction of these microRNAs may be a new approach to prevent and treat RNV.

## Figures and Tables

**Table 1:** Upregulated microRNAs in the OIR group.

MicroRNA	Base sequence	Normalized tag counts		Fold change	p value
		OIR	NOR		
mmu-miR-503-5p	UAGCAGCGGGAACAGUACUGCAG	129.33	72	1.80	0.01
mmu-miR-21a-5p	UAGCUUAUCAGACUGAUGUUGA	4521.67	2478.43	1.82	0.01

**Table 2:** Downregulated microRNAs in the OIR group.

MicroRNA	Base sequence	Normalized tag counts		Fold change	p value
		OIR	NOR		
mmu-miR-129-5p	AAGCCCUUACCCCAAAAAGCAU	75.67	175.33	0.43	0.01
mmu-miR-3099-3p	UAGGCUAGAGAGAGGUUGGGGA	24.00	56.00	0.43	<0.01
mmu-miR-150-5p	UCUCCCAACCCUUGUACCAGUG	42.33	96.00	0.44	<0.01
mmu-miR-129-3p	AAGCCCUUACCCCAAAAAGUAU	11.33	25.17	0.45	<0.01
mmu-miR-181c-3p	ACCAUCGACCGUUGAGUGGACC	218.33	920.67	0.24	0.01
mmu-miR-872-5p	UGAACUAUUGCAGUAGCCUCCU	35.67	79.26	0.45	<0.01
mmu-miR-181a-5p	AACAUUCAACGCUGUCGGUGAGU	7136.67	15514.50	0.46	<0.01
mmu-miR-129-5p	CUUUUUGCGGUCUGGGCUUGC	897.67	1870.14	0.48	<0.01
mmu-let-7b-5p	UGAGGUAGUAGGUUGUGUGGUU	566.00	1155.10	0.49	0.05
mmu-miR-383-5p	AGAUCAGAAGGUGACUGUGGCU	57.67	117.69	0.49	0.01
mmu-miR-369-3p	AAUAAUACAUGGUUGAUCUUU	115.33	230.67	0.50	0.01
mmu-miR-375-3p	UUUGUUCGUUCGGCUCGCGUGA	230.33	460.65	0.50	<0.01
mmu-miR-211-3p	GCAAGGACAGCAAAGGGGGGC	17.67	34.65	0.51	<0.01
mmu-miR-211-5p	UUCCCUUUGUCAUCCUUUGCCU	1224.67	2401.31	0.51	0.01
mmu-miR-490-3p	CAACCUGGAGGACUCCAUGCUG	34.00	66.67	0.51	0.01
mmu-miR-106b-5p	UAAAGUGCUGACAGUGCAGAU	197.33	386.92	0.51	0.05
mmu-let-7e-5p	UGAGGUAGGAGGUUGUAUAGUU	397.33	764.01	0.52	0.01
mmu-let-7c-5p	CUGUACAACCUUCUAGCUUUCC	10.33	19.87	0.52	0.01
mmu-miR-6540-5p	CUAAGGCAGGCAGACUUCAGUG	26.67	51.29	0.52	<0.01
mmu-miR-22-3p	AAGCUGCCAGUUGAAGAACUGU	387.00	744.23	0.52	0.01
mmu-miR-361-5p	UUAUCAGAAUCUCCAGGGGUAC	108.33	208.33	0.52	<0.01
mmu-miR-129-1-3p	AAGCCCUUACCCCAAAAAGUAU	19.00	35.84	0.53	0.01
mmu-miR-487b-3p	AAUCGUACAGGGUCAUCCACUU	169.67	316.36	0.53	0.04
mmu-miR-532-3p	CCUCCCACACCCAAGGCUUGCA	16.00	29.63	0.54	0.01
mmu-miR-423-3p	AGCUCGGUCUGAGGCCCCUCAGU	406.67	753.09	0.54	0.04
mmu-miR-320-3p	AAAAGCUGGGUUGAGAGGGCGA	146.00	270.36	0.54	0.03
mmu-miR-495-3p	AAACAAACAUGGUGCACUUCUU	70.00	127.27	0.55	0.02
mmu-miR-484	UCAGGCUCAGUCCCCUCCCGAU	225.67	402.98	0.56	0.02
mmu-miR-130a-3p	CAGUGCAAUGUUAAAAGGGCAU	40.33	72.01	0.56	<0.01
mmu-miR-181b-5p	AACAUUCAUUGCUGUCGGUGGGU	4193.00	7356.14	0.57	0.02
mmu-miR-409-3p	GAAUGUUGCUCGGUGAACCCCU	291.67	511.70	0.57	<0.01
mmu-miR-652-3p	AAUGGCGCCACUAGGGUUGUG	119.67	209.95	0.57	<0.01
mmu-miR-423-5p	UGAGGGGCAGAGAGCGAGACUUU	67.00	115.52	0.58	0.03
mmu-miR-130b-3p	CAGUGCAAUGAUGAAAGGGCAU	26.00	44.83	0.58	0.01
mmu-miR-127-5p	CUGAAGCUCAGAGGGCUCUGAU	513.33	870.05	0.59	<0.01
mmu-miR-34a-5p	UGGCAGUGUCUUAGCUGGUUGU	16.33	27.68	0.59	<0.01

**Table 3:** KEGG analysis of upregulated microRNAs in the OIR retina.

KEGG pathways	Fisher *p* value	Enrichment score	Genes
Apoptosis	0.02	1.79	ACTG1//HRK//MAP3K14
Ubiquitin-mediated proteolysis	0.02	1.76	KLHL13//KLHL9//RCHY1
Mitophagy	0.03	1.59	BNIP3L//FUNDC1
Tight junction	0.03	1.56	ACTG1//MYH1//PRKCE
p53 signaling pathway	0.03	1.54	PERP//RCHY1
Arrhythmogenic right ventricular cardiomyopathy (ARVC)	0.03	1.49	ACTG1//CACNB4
Hypertrophic cardiomyopathy (HCM)	0.04	1.36	ACTG1//CACNB4
Dilated cardiomyopathy (DCM)	0.05	1.32	ACTG1//CACNB4

**Table 4:** KEGG analysis of downregulated microRNAs in the OIR retina.

KEGG pathways	Fisher *p* value	Enrichment score	Genes
Ferroptosis	<0.01	2.54	PCBP1//PCBP2
PI3K-Akt signaling pathway	<0.01	2.36	CCND2//FASL//FGF18//NTF3
Ras signaling pathway	0.01	1.98	FASL//FGF18//NTF3
TGF-*β* signaling pathway	0.01	1.92	PITX2//SMAD7
MicroRNAs in cancer	0.02	1.78	CCND2//RECK//SPRY2
MAPK signaling pathway	0.02	1.73	FASL//FGF18//NTF3
Neurotrophin signaling pathway	0.02	1.63	FASL//NTF3
Cell cycle	0.02	1.61	CCND2//STAG2
FoxO signaling pathway	0.03	1.56	CCND2//FASL
Measles	0.03	1.54	CCND2//FASL
Hippo signaling pathway	0.04	1.44	CCND2//SMAD7

## Data Availability

The original data used to support the findings of this study are available from the corresponding authors upon request.

## Supplementary Materials

Supplementary Materials Supplementary Table 1: total RNA Quantification and Quality.
