# Supplementary material for: MicroRNA Expression Analysis of Mice Retinas with Oxygen-Induced Retinopathy by RNA Sequencing
Source: J Ophthalmol. 2022 Mar 3;2022:9738068. doi: 10.1155/2022/9738068 (PMC8913133; doi:10.1155/2022/9738068)
Supplement: Supplementary Materials — Supplementary Table 1: total RNA Quantification and Quality. [file 9738068.f1.docx]

| Sample Name | OD260/280 Ratio | OD260/230 Ratio | Conc. (ng/μl) | Volume (μl) | Total Amount (ng) |
| --- | --- | --- | --- | --- | --- |
| NOR1 | 1.82 | 2.53 | 1203.71 | 50 | 60185.5 |
| NOR2 | 1.83 | 2.5 | 1029.09 | 50 | 51454.5 |
| NOR3 | 1.89 | 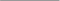   \| 2.53 \| \| --- \| | 1139.71 |    \| 30 \| \| --- \| | 34191.3 |
| OIR1 | 1.8 | 2.54 | 1278.1 | 50 | 63905 |
| OIR2 | 1.8 | 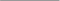   \| 2.55 \| \| --- \| | 939.98 |    \| 30 \| \| --- \| | 28199.4 |
| OIR3 | 1.81 | 2.52 | 1208.27 | 30 | 36248.1 |

Supplementary Table 1. Total RNA Quantification and Quality
